# Long-Term Supplementation of *Syzygium cumini* (L.) Skeels Concentrate Alleviates Age-Related Cognitive Deficit and Oxidative Damage: A Comparative Study of Young vs. Old Mice

**DOI:** 10.3390/nu15030666

**Published:** 2023-01-28

**Authors:** Nosheen Malik, Sana Javaid, Waseem Ashraf, Farhan Siddique, Muhammad Fawad Rasool, Faleh Alqahtani, Tanveer Ahmad, Muhammad Asad Abrar, Imran Imran

**Affiliations:** 1 Department of Pharmacology, Faculty of Pharmacy, Bahauddin Zakariya University, Multan 60800, Pakistan; 2 Department of Pharmacy, The Women University, Multan 60000, Pakistan; 3 Department of Pharmaceutical Chemistry, Faculty of Pharmacy, Bahauddin Zakariya University, Multan 60800, Pakistan; 4 Department of Pharmacy Practice, Faculty of Pharmacy, Bahauddin Zakariya University, Multan 60800, Pakistan; 5 Department of Pharmacology and Toxicology, College of Pharmacy, King Saud University, Riyadh 11451, Saudi Arabia; 6 Research Centre UGA/INSERM U1209/CNRS 5309, Institute for Advanced Biosciences, Grenoble Alpes University, 38000 Grenoble, France; 7 Drug Testing Laboratory Punjab, Multan 60800, Pakistan

**Keywords:** *Syzygium cumini*, dementia, Barnes maze, passive avoidance, antioxidants, aging

## Abstract

The *Syzygium cumini* (L.) Skeels is reported to have medicinal properties, but its benefits on age-related neurological changes have not been previously explored. In the current study, after phytochemical analysis of the pulp of *Syzygium cumini* (L.) Skeels fruit (*Sy. cmi*), young BALB/c mice have been supplemented with its 5, 15, and 30% dilution for 16 months, followed by behavioral experimentation and biochemical evaluation of isolated brains. The *Sy. cmi* has been found enriched with phenols/flavonoids while the occurrence of nine phytocompounds has been identified through GC-MS analysis. Further, *Sy. cmi* supplementation has caused significant (*p* < 0.05) protection from anxiety-like behavior in aged mice, and they have explored open, illuminated, and exposed areas of open field, light/dark, and an elevated plus maze, respectively. Furthermore, these animals have shown improved cognitive abilities as their percent (%) spontaneous alteration and novelty preference are significantly greater in T-maze and Y-maze and familiarity/novelty recognition tests. Further, *Sy. cmi*-supplemented mice remember the aversive stimuli zone and escape box location in passive avoidance and Barnes maze tests, and their brains have low levels of malondialdehyde and acetylcholinesterase with elevated antioxidant enzymes. The outcomes have provided scientific insight into the beneficial effects of *Sy. cmi* on age-associated amnesia that might be attributed to antioxidant and anticholinergic effects exerted by phytocompounds (caryophyllene, humulene, β-Farnesene, and phytol) owned by *Syzygium cumini*.

## 1. Introduction

Aging is a natural process involving anatomical, molecular, and vascular dynamics in different areas of the brain collectively contributing to the aging-associated decline in cognitive information [1]. Among various age-associated disorders, Alzheimer’s disease (AD) is the most prevalent type of dementia in late age that causes 60–80% of all cases of dementia [2]. It is a neurodegenerative disease with intracellular Ca^2+^ dysregulation, neurofibrillary tangles, phosphorylated tau protein accumulation, synaptic disruption, demyelination plaques, and beta-amyloid deposits in cerebral blood vessels in conjunction with the central cholinergic system deficits, which lead to progressive cognitive decline [3]. Further, the brains with AD have increased oxidative stress. Calcium is a renowned vital messenger that regulates a variety of physiological processes, which includes cell viability, programmed cell death, and synaptic plasticity. These pathways might be controlled by antioxidants by modulating the excessive intracellular free Ca^2+^ concentration, thus regulating the apoptosis and mitochondrial membrane depolarization processes.

The prevalence of neuropsychiatric events in the aged population is well established in various preclinical and clinical studies [4,5]. Around 98% of patients with dementia experience at least one neuropsychiatric illness, including anxiety and depression. The available therapeutic strategies to target AD are acetylcholinesterase (AchE) inhibitors, but the limited tolerability, poor bioavailability, and hepatotoxicity are challenging [6]. These available drugs targeting dementia are associated with adverse effects and usually stabilize the signs instead of alleviating them due to their short half-lives [7]. Therefore, there is a need for novel medications with better safety profiles. Alternatively, picking a healthy diet would be another preferable life-modifying intervention to reduce aging-related neurodegeneration and dementia while increasing life expectancy [8].

The pathogenesis of various age-related neurological disorders is triggered by increased oxidative stress as 2/3rd of the brain content is fat, which, along with its high oxygen requirements, makes it extremely vulnerable to oxidative damage [9]. With the advancement of age, an inequity develops between the brain’s antioxidant mechanism of defense and the oxygen species present within the cell [10]. High intakes of a diet enriched with antioxidants slow the declining effects of age on cognitive function. The antioxidants reduce amyloid deposition in the brain by modulating oxidative stress and inflammatory processes, which helps in the improvement of cognition in advanced age [11]. The fruit berries are antioxidant-rich, and dietary patterns incorporating them have delayed and even reversed the loss in cognitive functions related to age in laboratory animals [12]. Berry fruits include phytochemicals with anti-oxidative and anti-inflammatory properties that have a protective effect against brain aging and neurodegenerative illnesses [13]. Various polyphenols found in berries produce their neuroprotective effects through their capacity to penetrate blood-brain barrier (BBB), where their capability to scavenge free radicals and simultaneous stimulation of antioxidant enzymes work to reduce the aging-precipitated pathological levels of oxidative stress [13,14].

*Syzygium cumini* (L.) Skeels from the Myrtaceae family is vernacularly known as Jaman. It is cultivated in the sub-continent South Asia, in countries including Pakistan, and consumed as fruit. A variety of phytoconstituents, including anthocyanins, myricetin, glucoside, isoquercetin, ellagic acid, and kaemferol, are present in this plant [15]. The berries’ richness in polyphenols, sugar, and minerals enhances the health benefits of vitamin C, flavonoids, and anthocyanins. In traditional medicine, almost all portions of *Syzygium cumini* (L.) Skeels have been acknowledged as having various therapeutic characteristics. Its bark and fruit are reported to exert antihyperglycemic effects, making them an effective remedy for diabetes [16]. Further, a prominent protection from tissue damage has been noted in diabetic rat brains after oral administration of its seed extract for 6 weeks [17]. Moreover, the methanolic extract of *Syzygium cumini* exerts anticholinesterase as well as antioxidant effects in rats and mitigated scopolamine-induced spatial memory impairments [18].

This study has hypothesized that prolonged supplementation with *Syzygium cumini* (L.) Skeels fruit might protect from age-associated neurobehavioral impairment and brain oxidative stress. Initially, the fruit pulp has been chemically characterized for phytoconstituents by utilizing various in vitro assays. For in vivo experiments, juvenile BALB/c mice have been chronically nourished with freshly prepared dilutions of *Syzygium cumini* (L.) Skeels berry pulp to assess the impact of this nutritional supplementation on age-related anxiety and cognitive impairment in the geriatric mice model. Later, the isolated brains are further analyzed for the possible impact of this life-long dietary intervention on biochemical changes in the aged mice brains.

## 2. Materials and Methods

### 2.1. Sy. cmi Fruit Dilution Preparation

The fresh fruit of *Syzygium cumini* (L.) Skeels were locally purchased in July and rinsed with water to get rid of contaminants. After authentication by an expert botanist, the pulp was collected in the sterilized amber-colored container by de-seeding the fruit. The pulp was blended with water (1 kg pulp in 0.5 L tap water) to prepare a concentrate (*Sy. cmi*) and stored in non-reactive bottles in the freezer at −16 °C [19]. From the stock concentration of *Sy. cmi,* 5% *Sy. cmi*, 15% *Sy. cmi*, and 30% *Sy. cmi* dilutions were prepared.

### 2.2. Drugs and Chemicals

Drugs used in this study included donepezil and diazepam as Valium (10 mg/2 mL) from Roche Pharma, Karachi, Pakistan. These drugs and chemicals utilized in the biochemical analysis were of research grade.

### 2.3. Animals and Their Housing

Male BALB/c mice were bred locally and housed in the premises of the animal house of the Faculty of Pharmacy, Bahauddin Zakariya University, Multan. The housing conditions were maintained at a controlled temperature of 23 °C ± 2 °C and humidity maintained at 55–65% with a 12-h light/dark cycle. All animal studies received the approval of the Ethical Committee of the Department of Pharmacology BZU, Multan (08-PHL-S21) and instructions were followed by the “Institute of Laboratory Animal Resources” (ILAR), Commission on Life Sciences, National Research Council (NRC, 1996).

### 2.4. Animal Grouping and Their Diet

Healthy mice (*n* = 108) of 8–10 weeks were divided into six groups (*n* = 18). Three groups were fed with standard rodent chow and tap water while the remaining three groups were supplemented with *Sy. cmi* and water bottles replaced with bottles filled with *Sy. cmi* 5%, 15%, and 30% dilutions, respectively. All animals were housed as eight mice per cage (28 × 45 × 14 cm) with their particular beverage to age for 16 months.

**Figure 1:**
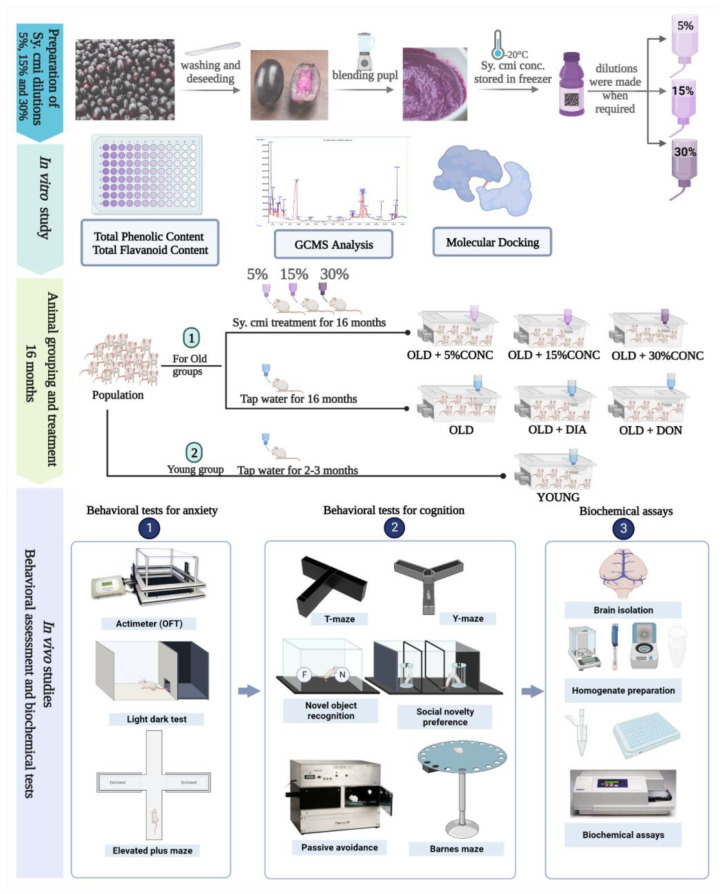
Experimental layout including preparation of *Sy. cmi* pulp dilutions with in vitro total phenols/flavonoids and Gas Chromatography-Mass Spectrometry (GC-MS)analysis. The illustrative depiction of animals grouping and supplementation with *Sy. cmi* dilutions for 16 months with subsequent neurobehavioral experimentation to evaluate the impact of *Sy. cmi* on (1) anxiety-like behavior and (2) age-related amnesia in mice followed by (3) biochemical analysis of isolated brains. Created with BioRender.com (XZ24R43BN4; accessed on 11 December 2022).

In detail, Group I was OLD AGED (used as geriatric control), Group II was OLD + DIA (treated with diazepam 1 mg/kg; intraperitoneally (i.p.) and used as a positive control in behavioral tests for anxiety), and Group III was OLD + DON (treated with donepezil 1 mg/kg; i.p. and utilized in behavioral tests for memory and learning as a positive control). Three test groups (Group IV–VI) were designated as the OLD + 5% *Sy. cmi* group, OLD + 15% *Sy. cmi* group, and OLD + 30% *Sy. cmi* group, which were used to evaluate the impact of *Syzygium cumini* (L.) Skeels on age-related neurobehavioral changes. Immediately before the behavioral experiments, a YOUNG group (used as young control) comprising 8–10-week-old mice was added. Animals in all groups were kept with their selected regimen until all behavioral assessments were completed. The whole animal grouping with scheme of study is summarized in Figure 1.

### 2.5. Behavioral Experiments

During the period of 16 months, few animals were found dead in the cage without any known reason. Thus, *n* = 8 from each group were finally included in behavioral experiments to maintain the homogeneity of group size. For acclimation, the test animals were transferred to the behavior room next to the housing room for at least an hour before the test. During behavioral testing for anxiolytic performance, the OLD + DIA group (*n* = 8) was treated with diazepam (1 mg/kg, i.p.) just 1 h before the test.

Before moving to the learning and memory tests, the OLD + DON group (*n* = 8) was administered donepezil (1 mg/kg, i.p.) for one week. The behavioral evaluation was conducted from 9:00 am to 6:00 pm and the apparatus was cleaned with ethanol 70% to remove any aromatic traces left by the preceding subject animal, and to eliminate any olfactory bias [20].

The Logitech Web Camera (Lausanne, Switzerland) and its software were used to capture animal motion during behavioral analysis. The camera for recording was held above each apparatus by using an elevated support system to record the animal’s activity in all zones of the apparatus. The test mice were unable to observe the experimenter while they were in the apparatus, which ensured that the mice’s behavior remained unaffected. The video recordings were then analyzed by using tracking software i.e., ANY-maze Video Tracking System (trial version 7.14 © 1999–2022 Stoelting Co, Wood Dale, IL 60191, USA).

### 2.6. Total Phenols and Flavonoid Content Estimation

The *Sy. cmi* was estimated for total phenolic content using a calibration curve, taking gallic acid in concentrations of 6.25, 12.5, 25, 50, 100, and 200 ug/mL as a standard reference according to the described method [21]. A total of 2 mg/mL of the sample taken in duplicates was mixed thoroughly with 10% (*v*/*v*) Folin–Ciocalteu reagent, followed by the addition of Na_2_CO_3_ (700 mM). After an incubation period of 2 h at 25 °C, absorbance was taken at 765 nm by using a microplate reader, and total phenols were estimated using the regression equation and reported as mg gallic acid equivalent per gram concentrate (mg GAE/g concentrate).

The total flavonoids present in *Sy. cmi* pulp were determined by an AlCl_3_ colorimetric method with slight modification [22]. The quercetin solutions of 12.5, 50, 100, 200, 400, and 800 ug/mL in concentrations were prepared in 95% methanol and were used to obtain the standard curve. A total of 50 µL of fruit pulp concentrate (2 mg/mL) and standard solutions of quercetin were added to 25 µL of 10% of the aluminum chloride solution, which was followed by 25 µL of methanol. 200 µL of (1 M) sodium acetate was added, which was followed by the addition of 200 µL distilled water to the mixture. The mixture was incubated at room temperature and protected from light for 40–50 min. The absorbance was noted at 415 nm; mean values of duplicates were used and results were expressed as mg quercetin equivalents per gram of pulp concentrate (mg QE/g concentrate).

### 2.7. Gas Chromatography-Mass Spectrometry (GC-MS)

An Agilent (7890B, Santa Clara, CA, USA) gas chromatograph equipped with an inert mass selective detector (5977B) and DB-5MS GC column (30 m in length, 0.25 mm in internal diameter, 0.25 µm in film thickness) was used for analysis. Sample injection (2 uL; methanolic fraction was prepared from fresh concentrate) was performed in the split-less mode, with an injector temperature of 250 °C and an interface temperature of 280 °C. The temperature of the oven was programmed from an initial value of 100 °C for 0.5 min andramped up to 340 °C at 20 °C/minute for 1 min. Helium was used as a carrier gas and electron impact ionization was used at −70 eV in full-scan mode. The run time was 30 min. Matching with National Institute of Standards and Technology (NIST) Library, Gaithersburg, MD 20899, USA, was carried out to identify the detected compounds.

### 2.8. Behavioral Assessment for Locomotor and Anxiolytic Activity

#### 2.8.1. Open Field Test (OFT)

To evaluate behavioral alterations in response to an exposed environment, the animal was permitted to roam in the open field apparatus. It measured rodents’ innate propensity for exploration in all zones of an open field. Each animal was placed in the center of the apparatus and its activity was assessed for 5 min in the open arena (45 × 45 cm) of an Actimeter (Panlab Harvard apparatus IR Actimeter, Barcelona, Spain) equipped with two frames, comprising 16 photobeam sensors to detect the locomotor activity of mice [23]. Simultaneously, the anxiolytic activity was assessed by monitoring the animal’s number of entries in the central zones and the amount of time spent there. The vertical locomotor activity i.e., rearing in terms of slow rearing (SR) and fast rearing (FR), was measured by counting the interruptions in the photobeam of the upper frame, and horizontal locomotion in terms of the slow movement (SM) and fast movement (FM) was counted by the interruptions in the photobeam of the lower frame with the help of the control unit.

#### 2.8.2. Light-Dark (LD) Test

This test evaluated the impact of *Sy. cmi* supplementation on anxiety in mice. Using a two-compartment box consisting of a light chamber (21 × 21 × 25 cm) connected by a small opening (7 × 6 cm) to a dark chamber (20 × 40 × 40 cm), the mice were introduced into the light chamber and allowed free crossings between both chambers for 5 min. The number of visits and duration in the light chamber were recorded to note the animal’s preference for the light chamber, which was taken as an indicator of reduced anxiety [24].

#### 2.8.3. Elevated-Plus Maze (EPM) Test

Mice were tested in a plus-shaped maze with two open and two closed arms (15 × 5.5 cm) positioned 45 cm above the floor [25]. Mice, by nature, prefer to reside in enclosed dark spaces, but also make the struggle to explore exposed areas. The mice were allowed to explore all four arms for 5 min after being introduced to the maze by being placed in front of the open arm at the beginning of the test. To evaluate the inter-group difference for anxiety, the entries and duration of each animal were noted in the open arms, as the preference for the open arena was taken as an indication of anxiolytic behavior [26].

### 2.9. Behavioral Tests for Learning and Memory

#### 2.9.1. T-Maze (Spontaneous Alteration) Test

The three arms (30 × 10 cm) comprising the T-maze were used to estimate the impact of *Sy. cmi* on the age-related cognitive incapacity of mice. Animals began at the base of an arm (A-arm) and freely chose one of the adjacently located two target arms (B and C arms) during the test duration of 5 min. The animal’s urge to explore its novel surroundings was shown as the animal’s alternation behavior i.e., ABC, BAC, CAB, etc. The rodent typically selected the arm that hadn’t been visited previously while remembering the prior selection, which induced spontaneous alternation [27], which was calculated by the following formula:

(1) $$\%\;\mathrm{Spontaneous}\;\mathrm{alternation}\;=\frac{\left(\mathrm{Alternations}\;\mathrm{number}\right)\;}{\left(\mathrm{Total}\;\mathrm{entries}\;\mathrm{in}\;\mathrm{all}\;\mathrm{arms}-2\right)}\times 100$$

#### 2.9.2. Y-Maze (Novel Arm) Test

The test was carried out in a Y-shaped maze comprising three similar arms (40 × 8 × 15 cm each) arranged at 120°. The Y-maze test had two trials of 5 min carried out at an inter-trial interval of 24 h. The first trial was designated as a training session in which two arms were kept open and a novel arm was barricaded with a slit. After 24 h of the training session, all three arms were kept open, and mice were tested for 5 min in order to note the animal’s preference for the novel arm in terms of the duration of novel and familiar arms exploration to calculate the novel arm preference index [28,29].

(2) $$\mathrm{Novel}\;\mathrm{arm}\;\mathrm{preference}\;\mathrm{Index}\;=\frac{\left(\mathrm{Novel}\;\mathrm{arm}\;\mathrm{duration}\right)\;}{\left(\mathrm{Novel}\;\mathrm{and}\;\mathrm{familiar}\;\mathrm{arms}\;\mathrm{duration}\right)}\times 100$$

#### 2.9.3. Novel Object Recognition Test

This test used the intrinsic propensity of rodents to connect with unfamiliar surroundings, as they prefer to spend more time investigating the novel object. Thus, the rodent’s memory was tested by estimating its capacity to differentiate between unfamiliar and familiar objects [30]. Every animal was exposed to two consecutive sessions carried out in a square area (40 × 40 cm) with high walls (38 cm). In the training session, two identical objects were put down and mice were given 10 min to investigate these two similar objects [31]. In a test session, one of the previously investigated objects was replaced by a novel object, and the animals’ interactions with both objects were observed. The results were used to compute the discrimination index (DI), which, if increased, indicated that the animal had improved working memory. The DI was computed by using the formula:

(3) $$\mathrm{Discrimination}\;\mathrm{Index}\;=\frac{\left(\mathrm{Novel}\;\mathrm{object}\;\mathrm{duration}\;-\mathrm{Familiar}\;\mathrm{object}\;\mathrm{duration}\right)\;}{\left(\mathrm{Novel}\;\mathrm{object}\;\mathrm{duration}+\mathrm{Familiar}\;\mathrm{object}\;\mathrm{duration}\right)}\times 100$$

#### 2.9.4. Social Novelty Preference Test

This test was used to evaluate the subject animal’s memory to distinguish the new animal from the one with which it was previously engaged. The equipment consisted of a three-chambered rectangular box (20 × 40 × 47 cm) in which the right and left chambers had spherical wire cages (diameter 9 cm, height 11 cm, with vertical bars spaced apart by 0.5 cm) [23]. In the training phase of 10 min, two conspecific animals were introduced in spherical cages, and test animals were individually allowed to interact with these two mice. Subsequently, one of these mice was replaced with a novel one and a test session of 5 min was conducted in which animals were tested for their ability to differentiate the novel from familiarized mice. The interactions with the novel and familiarized mice were noted and the social novelty preference index was calculated as follows [32].

(4) $$\mathrm{Social}\;\mathrm{novelty}\;\mathrm{preference}\;\mathrm{Index}\;=\frac{\left(\mathrm{Novel}\;\mathrm{animal}\;\mathrm{duration}\;-\mathrm{Familiar}\;\mathrm{animal}\;\mathrm{duration}\right)\;}{\left(\mathrm{Novel}\;\mathrm{animal}\;\mathrm{duration}+\mathrm{Familiar}\;\mathrm{animal}\;\mathrm{duration}\right)}\times 100$$

#### 2.9.5. Passive Avoidance (Step-Through) Test

The Gemini avoidance system, comprising inter-connected chambers (light and dark), was used to estimate the animal’s long-term memory. The test comprised one acquisition trial and two retention trials. During the acquisition trial, the animal was placed individually in a light compartment, and after 30 s of acclimation, the door was opened for 150 s, which allowed the animals to leave the lightened area and enter the dark chamber, due to their innate preference for dark places. Soon after they entered the dark chamber, a 0.3 mA electric shock was delivered for 2 s. The retention trials were held 60 min (1st retention trial) and 24 h (2nd retention trial) after the acquisition trial in which the animal was placed in the light chamber and permitted to explore both chambers for 5 min to note their step-through latencies [33].

#### 2.9.6. Barnes Maze Test

In the Barnes maze test, the animals were encouraged to cross an open platform surface and enter a small, dark compartment beneath the platform through a hole designated as an escape hole. This experiment was conducted on an open circular platform with a 92 cm diameter that was 90 cm above the ground and surrounded by fixed extra cues to aid mice in navigation in the Barnes maze. The circular area of the apparatus comprised 20 uniformly scattered holes, each 5 cm in diameter and an escape box (28 × 22 × 21 cm) that was placed under one specific hole, the target hole, which was kept constant throughout the study. The vast platform arena of the maze acted aversively for mice in this test because rodents naturally prefer a dark, calm environment. A mild aversive stimulus of noise was also used to generate an unpleasant environment that would encourage mice to leave the circular platform. However, this stimulus was not extremely hostile so as not to distract the animal’s attention away from the spatial task [34]. Day 1 of the test was the habituation phase, in which mice were given 60 s to explore the maze and then were carefully introduced into the escape box through the escape hole, where the animals were kept for 3 min. In case of an animal’s resistance to enter the target hole, the base of the tail was gently pulled away, as this causes mice to enter the hole [35]. From test days 2–5, animals were individually introduced into the middle of the circular maze and allowed to move and explore the maze and holes for 180 s, unless it tracked down the escape box hole. If mice entered the target hole, it was allowed to stay there for 30 s. If within 180 s mice didn’t enter the escape hole, they were gently coaxed into the hole and given 2 min to remain there. During test days, there were four trials per day for each animal, which were conducted with a 30 min inter-trial break, and their latencies to enter the target hole were noted [34]. On day 6 of the test, a probe trial of 90 s was carried out with no escape box, and animals were tested for their capability to remember the exact location of the target hole. A camera was mounted on the ceiling, which recorded the locomotor activity, which helped in the estimation of the animals’ latency (s) to reach the target hole during acquisition days, the number of pokes in the target hole, the time of escape hole exploration, and the distance traveled to find the escape hole on probe day [23].

### 2.10. Preparation of Brain Homogenates for Biochemical Analysis

After the Barnes maze test, the randomly selected mice (*n* = 6) from all groups were decapitated to isolate their brains. 0.3 g of each isolated brain was homogenized in 3 mL of phosphate buffer (pH 7.4) using a homogenizer (IKA, Tech), and the resulting homogenate was centrifuged at 12,000 rpm for 10 min at 4 °C. The supernatant was removed and divided into aliquots which were stored at −40 °C until further analysis [26] for malondialdehyde, superoxide dismutase, acetylcholinesterase, glutathione peroxidase, and catalase assays. The outcomes were normalized by protein content, which was quantified by the previously described Lowry’s method [36].

#### 2.10.1. Malondialdehyde (MDA) Assay

To estimate the impact of *Sy. cmi* on age-related lipid peroxidation, freshly prepared 15% trichloroacetic acid (TCA) and 0.37% thiobarbituric acid (TBA) were mixed with brain homogenates in a 1:1 ratio [37]. The reaction mixture was boiled (100 °C) for about 15 min and then cooled (room temperature). The reaction mixtures were centrifuged at 3500 rpm for 10 min at 4 °C and loaded in a 96-well microplate to note the absorbance at 532 nm, and MDA levels were calculated as nmol/mg of protein by using its extinction coefficient (1.56 × 105 M^−1^ cm^−1^).

#### 2.10.2. Superoxide Dismutase (SOD) Assay

Brain homogenates were combined with 50 mM sodium carbonate, 24 µM of nitro blue tetrazolium (NBT), and 0.1 mM of ethylenediaminetetraacetic acid (EDTA)(Sigma Aldrich, St. Louis, MO, USA). The reaction mixtures were loaded in the 96-well plate (each sample was loaded in duplicate), followed by the addition of 1 mM of hydroxylamine HCl to initiate the reaction. The absorbance was noted every 5 min up to 20 min at 570 nm, and activity was evaluated and expressed as units of SOD/mg of protein [38].

#### 2.10.3. Glutathione Peroxidase (GPx) Assay

An amount of 0.8 mM EDTA, 10 mM sodium azide, 2.5 mM H_2_O_2_, 0.1 M reduced glutathione, and phosphate buffer were added to each brain homogenate, and mixtures were incubated (37 °C) for 15 min [38]. To end the reaction, 10% TCA was added, followed by centrifugation at 4 °C for 5 min using 15,000 rpm. The supernatant was separated and 0.3 mM disodium hydrogen phosphate and 0.04% DTNB were added, and absorbance at 412 nm was noted in a microplate reader. The extinction coefficient 14.15 mM^−1^ cm^−1^ was used to calculate Gpx activity and expressed in nmol/min/mg of protein.

#### 2.10.4. Catalase (CAT) Assay

Amounts of 5% K_2_CR_2_O_7_-acetic acid solution (1:3 by volume) and 0.2 M H_2_O_2_ solution were prepared freshly. The mixture comprising tissue homogenate, phosphate buffer, and H_2_O_2_ was incubated (37 °C) for 90 s and followed by the addition of dichromate acetic acid reagent, which stopped the reaction and changed the color to blue. This mixture was boiled (100 °C) for about 15 min, which changed the reaction mixture color to green. After centrifugation at 4 °C at 2500 rpm for 5 min, the absorbance was noted in the microplate reader (Spectramax 340 PC384 by Molecular Devices, CA, USA) at 570 nm. The blank and standard were simultaneously run with the test brain homogenates, and the catalase activity was measured using the 43.6 M^−1^ cm^−1^ as the extinction coefficient for H_2_O_2_. Outcomes were expressed as µmol/min/mg of protein [39].

#### 2.10.5. Acetylcholinesterase (AChE) Assay

The acetylcholinesterase levels in brain homogenates were determined by the previously described method [40]. Brain homogenate sample, 0.01 M 5,5′-dithio-bis-2-nitrobenzoic acid (DTNB), and phosphate saline buffer were mixed, keeping the temperature at 25 °C, and absorbance was noted at 412 nm. Then, 0.075 M solution of acetylthiocholine iodide was added, and a change in absorbance was noted at 412 nm every 2 min for 10 min to calculate the AchE activity as µmol/min/mg of protein using ɛ = 13.6 mM^−1^ cm^−1^.

### 2.11. Molecular Docking Methodology

#### 2.11.1. Selection of Protein Targets

To execute the virtual screening process, the crystal structure of acetylcholinesterase was downloaded from the Research Collaboratory for structural bioinformatics (RCSB) protein data bank with the Protein Data Bank (PDB) ID: 1GQR [41].

#### 2.11.2. Softwares Required

For molecular docking, we utilized MGLtools, the Autodock4 and Autogrid4 [42] binary files, the Discovery Studio Visualizer from BIOVIA (San Diego, CA, USA) [43], ChemDraw ultra [44], and ChemDraw 3D pro [45].

#### 2.11.3. Preparation of Protein

This protein was obtained from RCSB and then processed so that the autodock suite could be run on it. In BIOVIA’s discovery studio visualizer, we eliminated all heteroatoms as well as co-crystal ligand and solvent molecules from the protein molecule. After obtaining a clean protein structure, autodock tools were used to optimize it for docking by giving each atom the appropriate polar hydrogen and Kollman charges and saving the file as a pdbqt [46].

#### 2.11.4. Preparation of Ligand and Molecular Docking

Using the compounds’ International union of pure and applied chemistry (IUPAC) designations, the structures were drawn in ChemDraw ultra, and then the energy minimization was performed in chem 3D pro. Spatial data files (SDF) with the compound structures were stored. After that, the openbabel graphical user interface (GUI) program was used to transform these structures into a pdbqt file, which is a format that autodock can read. Autodock4 was then used to do the structure-based virtual screening. For creating the grid parameter file, the box dimensions were retained the same as for the co-crystal ligand. Docking parameter files were generated using a combination of a custom force field called Autodock4Zn and a Lamarckian genetic algorithm (LGA). In order to guarantee the highest level of accuracy, we used a population size of 300 and a number of postures of 50 [47]. Following preparation, the ligand library was docked independently into the active site of each protein [42].

#### 2.11.5. Visualization

For this purpose, we employed the ligand-protein interaction analyzer BIOVIA discovery studio visualizer. Protein pdbqt and autodock output files were imported into BIOVIA’s Discovery Studio to create all the 2D and 3D conformations. It was determined which contacts between the ligand and active pocket were bonding and which were not.

#### 2.11.6. Validation

The RMSD value and re-docking of the co-crystal ligand into the active pocket of the protein were used to verify the docking technique. Docking and experimental ligand RMSD values less than 2.0 were required for the acceptance of poses [48].

### 2.12. Statistical Analysis

The results were evaluated by using GraphPad Prism version 8 (San Diego, CA, USA). All behavioral and biochemical parameters were evaluated by parametric one-way ANOVA followed by Dunnett’s multiple comparison test, except step-through latencies and escape latencies in passive avoidance, and the Barnes maze was evaluated by two-way ANOVA. Data were shown as mean ± standard error of mean (SEM) for *n* = 8 in behavioral experiments and *n* = 6 in biochemical evaluation tests taking *p* < 0.05 as significant. *p* < 0.05 was considered significant statistically.

## 3. Results

### 3.1. Total Phenols and Flavonoids Content

The results have revealed that per gram of *Sy. cmi* concentrate is equivalent to 28.21 ± 8.3 mg of gallic acid in the estimation of total phenolic content and 6.32 ± 0.9 mg of quercetin in the estimation of the total flavonoid content.

### 3.2. GC–MS Analysis for Phytocomponents

**Figure 2:**
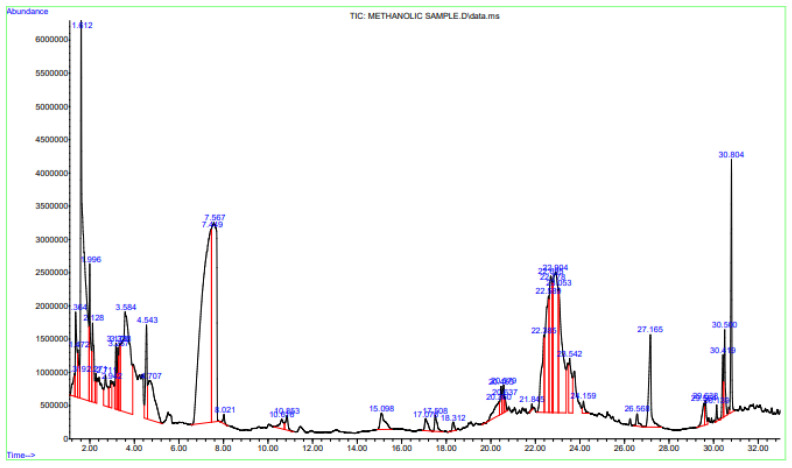
Total ion chromatogram (TIC) of *Syzygium cumini* (L.) Skeels. The lines in chromatogram represent different compounds along with their respective retention time.

GC–MS analysis shows the presence of acyclic monoterpenoids like ocimene, acyclic diterpenoids like phytol, sesquiterpenoids like caryophyllene, humulene, and farnesene, cyclic aldehydes like 5-hydroxymethylfurfural, and fatty acids like dodecanoic acid and oleic acid (Figure 2). The retention time of the respective compounds and their relative percentage of area is reported in Table 1.

### 3.3. Impact of Sy. cmi on Anxiety-like Behavior and Locomotor Activity in an Open Field

**Figure 3:**
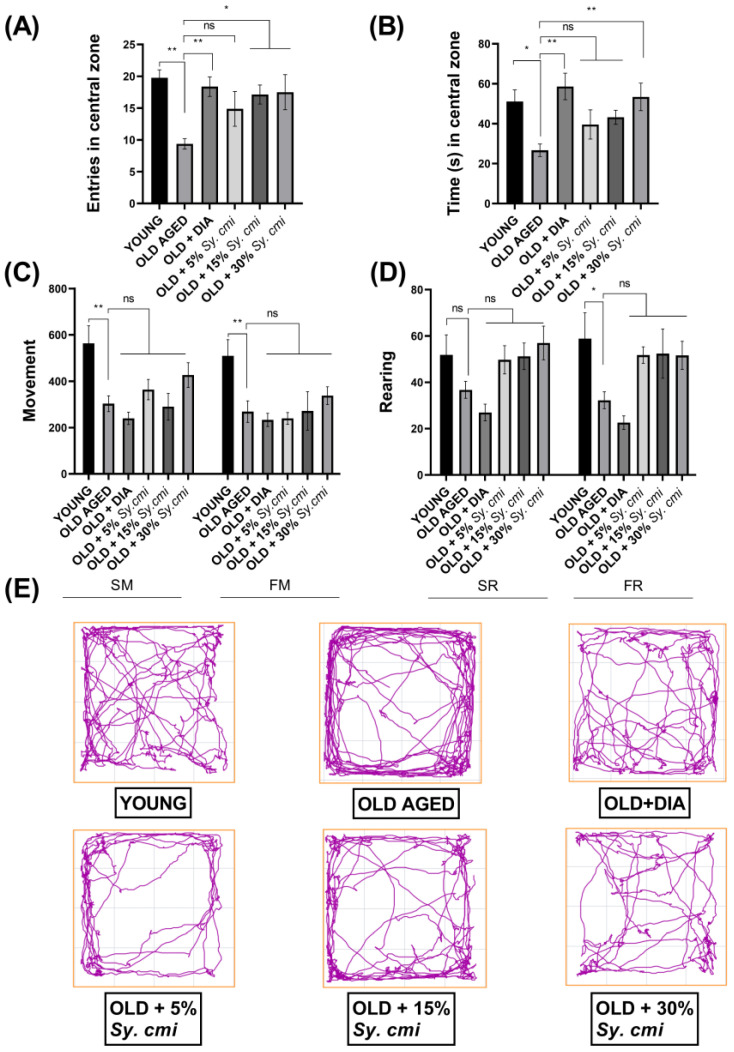
The mice (*n* = 8) supplemented with 5, 15, and 30% dilutions of *Sy. cmi* for 16 months were allowed to explore the square-shaped arena for 5 min to evaluate anxiety-like behavior by noticing their (**A**) entries in central zone, and (**B**) time in central zone. Further, the mice were examined for their horizontal and vertical locomotor activity and outcomes were expressed as (**C**) movements, (**D**) rearing, and (**E**) track plots of animal activity in open field. The outcomes of all groups were compared with the aged control group and * *p* < 0.05, ** *p* < 0.01 were considered statistically significant while ns showed non-sgnificant outcomes for all data presented as mean ± SEM.

In OFT, one-way ANOVA has shown a notable difference in the number of entries and duration spent in the central zone among all groups with (F (5,42) = 3.73, *p* = 0.007) and (F (5,42) = 3.93, *p* = 0.005), respectively. In detail, the old mice dwell less (*p* = 0.001) in the central zone than the young mice, depicting that they are frightened of exposed areas, showing age-related anxiety-like behavior in older mice. Further, the elderly mice remain restricted to the peripheries of the maze and spend maximum time along the walls, resulting in a significantly shorter duration (*p* = 0.020) in the center. However, this age-related anxiousness has been attenuated by nourishing the young mice with 15% and 30% *Sy. cmi* for chronic phases of their life span, as their entries in the central zone are increased with *p* = 0.027 and *p* = 0.019, respectively, as compared to old-age mice consuming only standard food as shown in Figure 3A. The mice receiving 30% *Sy. cmi* have spent the significantly longer duration in an open arena (*p* = 0.009) as compared to older untreated animals, same as the diazepam did (*p* = 0.0016) (Figure 3B). However, the observed outcomes remain non-significantly different in 5% *Sy. cmi* treated mice.

As two of the behaviors involved in locomotion are exploring and walking in open field activity, the mice have also been observed for their vertical and horizontal locomotion in terms of the number of slow and fast rearing and movements. A marked decrease in horizontal movements is noted in old-aged mice as compared to young animals for slow movements (*p* = 0.003) and fast movements (*p* = 0.007), and older mice prefer to sit in the outer corners of the maze with less exploration of the center. Likewise, fast rearing is decreased in geriatric mice (*p* = 0.025) as compared to young animals. However, the impact of *Sy. cmi* supplementation does not reverse the age-related decline in locomotor activity in mice, as the difference for movements and rearing remains non-significant (*p* > 0.05) in *Sy. cmi* treated groups as compared to older untreated mice (Figure 3C,D).

### 3.4. Impact of Sy. cmi on Anxiety-like Behavior in Light/Dark and ElevatedPlus-Maze Tests

**Figure 4:**
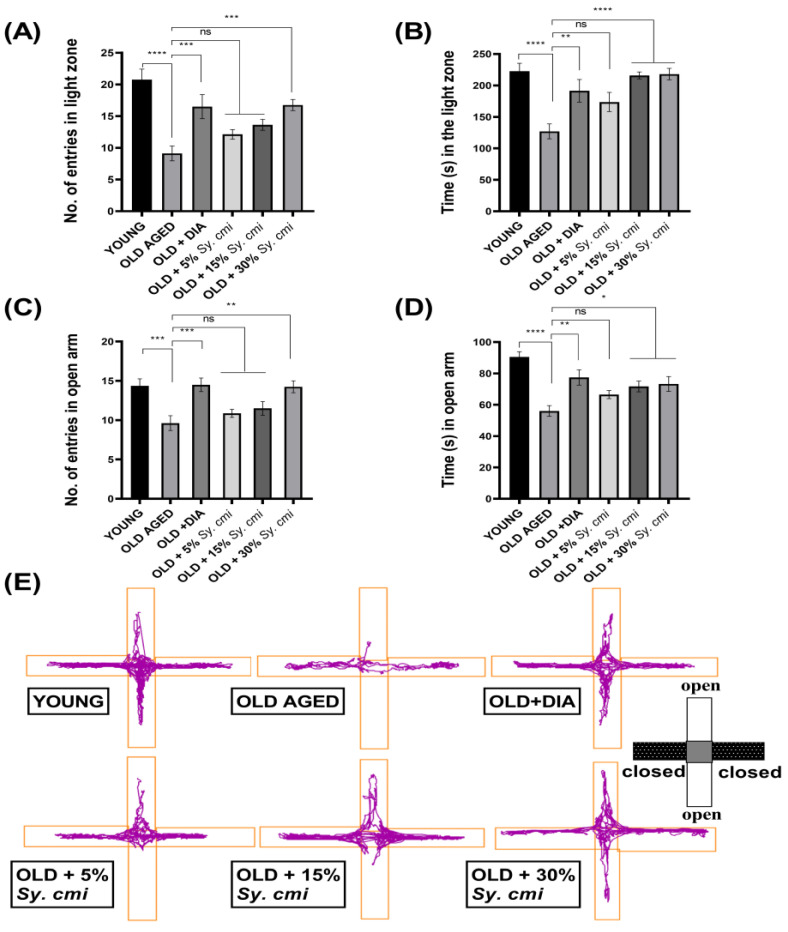
The mice (*n* = 8) supplemented with 5, 15, and 30% dilutions of *Sy. cmi* for 16 months were tested for anxiety-like behavior in light/dark and elevated plus-maze tests, and their exploration of both mazes for 5 min was evaluated for (**A**) number of entries in light zone, (**B**) time in light zone, (**C**) number of entries in open arms, (**D**) time in open arms, and (**E**) representative track plots of animal’s activity in the elevated plus-maze. The outcomes of all groups were compared with the aged control group and * *p* < 0.05, ** *p* < 0.01, *** *p* < 0.001, **** *p* < 0.0001 were considered statistically significant while ns showed non-sgnificant outcomes for all data presented as mean ± SEM.Moreover, animals have been further tested for their entries and duration of stay in the open and closed arms of the EPM. The outcomes of ANOVA reveal a significant difference among groups for entries in open arms (F (5,42) = 6.74, *p* = 0.0001) and time spent there (F (5,42) = 9.10, *p* < 0.0001). In comparison to young mice, the older untreated mice show noticeably fewer entries in open arms (*p* = 0.0008) and duration of open-arm exploration (*p* < 0.0001). However, this increase in age-related anxiety-like behavior has been ameliorated in mice chronically treated with *Sy. cmi*. As compared to geriatric mice, nourishment with 30% *Sy. cmi* results in increased entries in open arms with *p* = 0.001 (Figure 4C), and these mice explore the open arms for a longer duration with *p* = 0.011 (Figure 4D). The outcomes with 15% *Sy. cmi* are also noticeable in terms of duration of stay in open arms with *p* = 0.024, but the observed parameters remain non-significantly changed in mice administered with 5% *Sy. cmi*.

In the L/D test, animals are noted for their preference for the light zone, and one-way ANOVA describes a significant inter-group variation for the number of visits (F (5,42) = 10.04, *p* < 0.0001) and duration (F (5,42) = 8.41, *p* < 0.0001) in the light zone. As compared to young mice, aged mice enter less in the light zone (*p* < 0.0001) and spend less time there (*p* < 0.0001), suggesting age-related anxiety-like behavior. This age-related fear of the illuminated arena has been ameliorated by 30% dilutions of *Sy. cmi* as increased entries (*p* = 0.0007) and duration (*p* < 0.0001) in the lighted area are noted (Figure 4A,B). The old animals provided with 15% *Sy. cmi* also stay for a longer time (*p* < 0.0001) in the light zone as compared to aged untreated mice.

### 3.5. Impact of Sy. cmi on Learning and Memory in T-Maze and Y-Maze Tests

**Figure 5:**
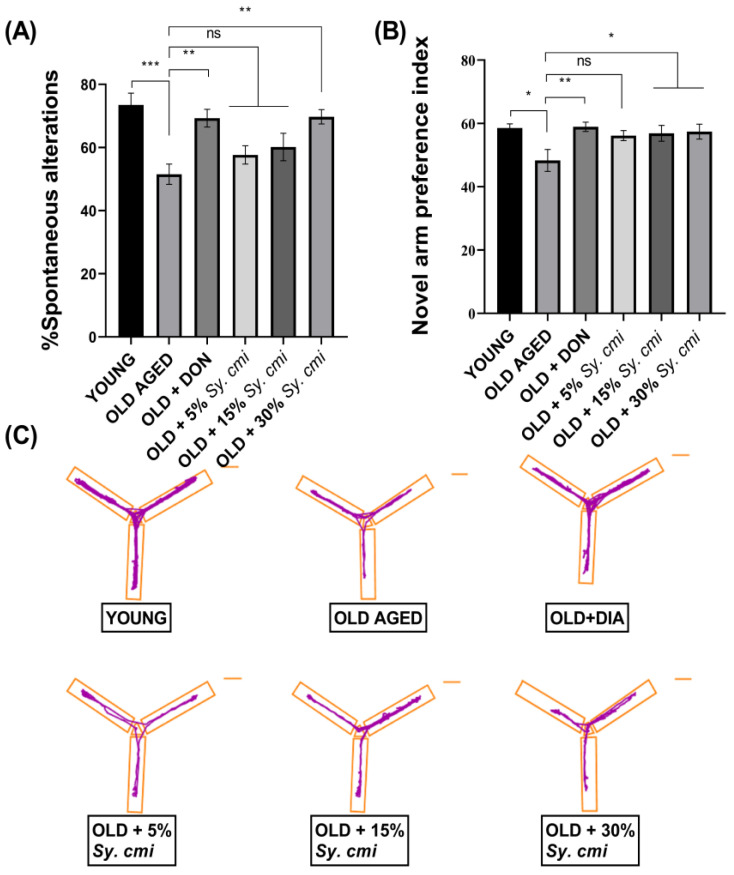
The mice (*n* = 8) supplemented with 5, 15, and 30% dilutions of *Sy. cmi* for 16 months were tested for learning and memory in T-maze and Y-maze tests, and their activity in the maze for 5 min was evaluated for (**A**) % spontaneous alteration, (**B**) novel arm preference index, and (**C**) representative track plots of animals’ activity in the Y-maze. The outcomes of all groups were compared with the aged control group and * *p* < 0.05,** *p* < 0.01, *** *p* < 0.001 were considered statistically significant while ns showed non-sgnificant outcomes for all data presented as mean ± SEM.

For the evaluation of working memory, mice have been tested in alternation tasks and their disposition to explore novel milieus has been monitored. The T-maze spontaneous alternation test is used to assess the impact of *Sy. cmi* on short-term spatial memory in older mice. The ANOVA shows a considerable variation in alternation behavior among differently treated mice (F (5,42) = 6.74, *p* = 0.0001). In comparison to young animals, age-dependent amnesia has been observed in older mice as their spontaneous alteration is significantly (*p* = 0.0001) reduced. The 30% *Sy. cmi* treated mice show improved recollection of previously visited arms of the maze, as increased spontaneous alternation behavior is noted in these animals (Figure 5A). These mice show reduced age-related amnesia as their % SAP score is higher (*p* = 0.0014) in comparison to old untreated mice, similar to the outcomes noted in the donepezil-treated group (*p* = 0.0019).

In the y-maze test, the novel arm preference index varies significantly among all groups with (F (5,42 = 3.03, *p* = 0.019). The 18–19 months old mice show reduced remembrance of the previously visited zone of the y-maze, as they explore the novel arm for a significantly shorter duration, resulting in their reduced preference index (*p* = 0.010) compared to young mice. Anyhow, the mice supplemented with *Sy. cmi* show concentration-dependent improvement in the recognition of previously explored arms, as older mice receiving 15% and 30% *Sy. cmi* prefer the novel arm over the familiar one, and their novel arm preference index is significantly more than aged control with *p* = 0.041 and *p* = 0.028, respectively (Figure 5B).

### 3.6. Impact of Sy. cmi on Learning and Memory in Familiarity/Novelty Preference Tests

**Figure 6:**
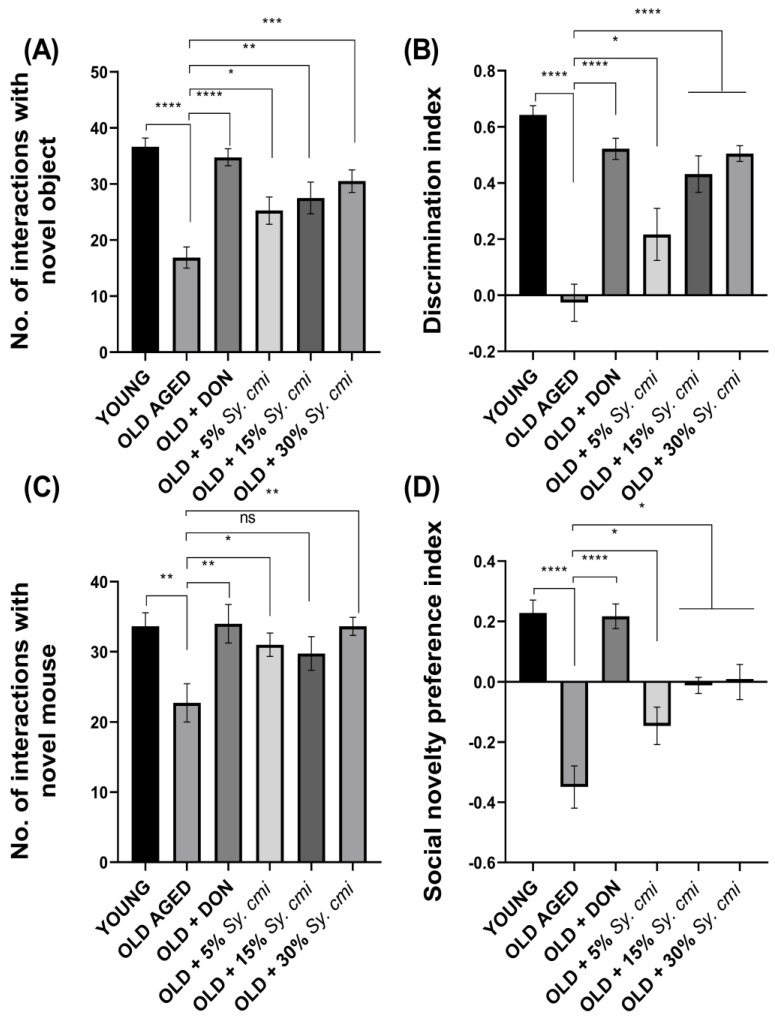
The mice (*n* = 8) supplemented with 5, 15, and 30% dilutions of *Sy. cmi* for 16 months were tested for learning and memory in novel object and social recognition tests, and the animals’ cognition was tested by monitoring their remembrance of familiarity during a test phase of 5 min duration, and outcomes were expressed as (**A**) number of interactions with the novel object, (**B**) discrimination index, (**C**) number of interactions with the novel mouse, and (**D**) social novelty preference index. The outcomes of all groups were compared with the aged control group and * *p* < 0.05, ** *p* < 0.01, *** *p* < 0.001, **** *p* < 0.0001 were considered statistically significant while ns showed non-sgnificant outcomes for all data presented as mean ± SEM.

The results of ANOVA reveal that all groups vary significantly for the number of interactions with a novel object (F (5,42) = 11.68, (*p* < 0.0001)) and discrimination index (F (5,42) = 17.49, (*p* < 0.0001)) during the test trial of the novel object recognition test. In detail, despite familiarization with identical objects, the elderly mice show a poor capability to discriminate the familiar object from the novel one, showing the inappropriate remembrance capacity of the aged group. During the test trial, their interactions with novel object exploration are notably less (*p* < 0.0001), resulting in a reduced discrimination index (*p* < 0.0001) as compared to young mice. While long-term supplementation with *Sy. cmi* causes aged mice to improve their recollection of the familiarized object, they more frequently sniff and interact with the novel object (*p* = 0.029) in a concentration-dependent manner (Figure 6A). Similarly, the discrimination index is notably enhanced (*p* = 0.022) in animals treated with *Sy. cmi* (Figure 6B).

Additionally, the mice have been examined for their memory by evaluating their memory of previously familiarized animals during the social interaction test. The outcomes of ANOVA reveal that age exerts a significant impact on animal recognition memory, as noteworthy inter-group variation is noted for the number of interactions (F (5,42) = 3.80, (*p* = 0.0063)) and social novelty preference index (F (5,42) = 17.74, (*p* < 0.0001)). The aged mice show reduced discrimination capability as they interact less with the novel animal as compared to the young group (*p* = 0.005) (Figure 6C), and the social novelty preference index is also reduced in these animals (*p* < 0.0001) (Figure 6D). However, the mice chronically nourished with *Sy. cmi* have less impact of age on their recognition ability as they approach and sniff novel mouse more frequently (*p* = 0.046), leading to an increased novelty preference index (*p* < 0.036) in comparison to old untreated mice.

### 3.7. Impact of Sy. cmi on Learning and Memory in Passive Avoidance Test

**Figure 7:**
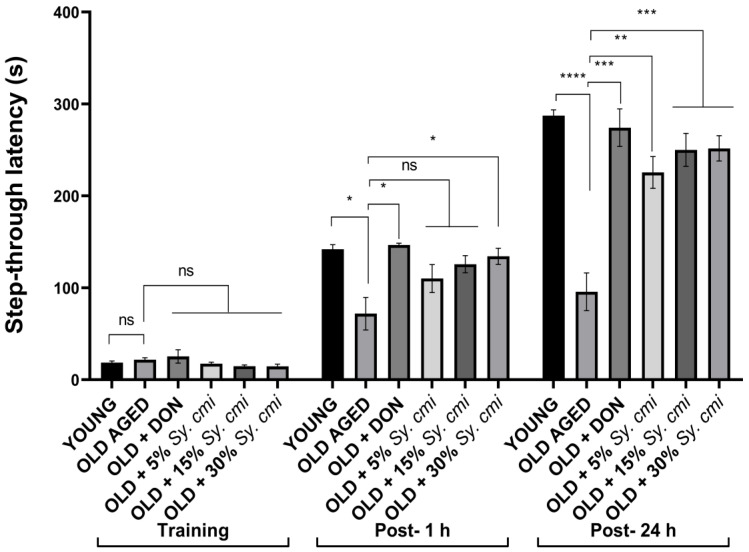
The mice (*n* = 8) supplemented with 5, 15, and 30% dilutions of *Sy. cmi* for 16 months were tested for learning and memory in a passive avoidance test. The animals’ cognitive ability was tested by monitoring their remembrance of aversive stimuli in two sessions of 5 min duration carried out after 1 h and 24 h of the training session, and avoidance of aversive stimuli zone was expressed as step-through latency. The outcomes of all groups were compared with the aged control group and *p < 0.05, ** *p* < 0.01, *** *p* < 0.001, **** *p* < 0.0001 were considered statistically significant while ns showed non-sgnificant outcomes for all data presented as mean ± SEM.

The results of two-way ANOVA show a significant result in step-through latencies (F (42,84) = 17.13, (*p* < 0.0001)) among all groups when compared to the control group. The young mice remember the aversive stimuli that have been presented in the dark compartment, as they show increased latencies to enter the dark room in both test trials. The aging causes poor recalling abilities in mice, as the aged control group has shorter step-through latencies in post-1 h (*p* = 0.018) and post-24 h (*p* < 0.0001) retention trials. The long-term supplementation with 30% *Sy. cmi* causes improvement in memory of shock stimuli, as these animals show significantly increased latency (*p* = 0.037) to enter the dark compartment. However, concentration-dependent results are observed in the post-24 h trial, as *Sy. cmi* treatment causes longer latencies to enter the dark, i.e., 5% (*p* = 0.001), 15% (*p* = 0.0003), and 30% (*p* = 0.0002) in comparison to the aged control group as shown in Figure 7.

### 3.8. Impact of Sy. cmi on Learning and Memory in Barnes Maze Test

**Figure 8:**
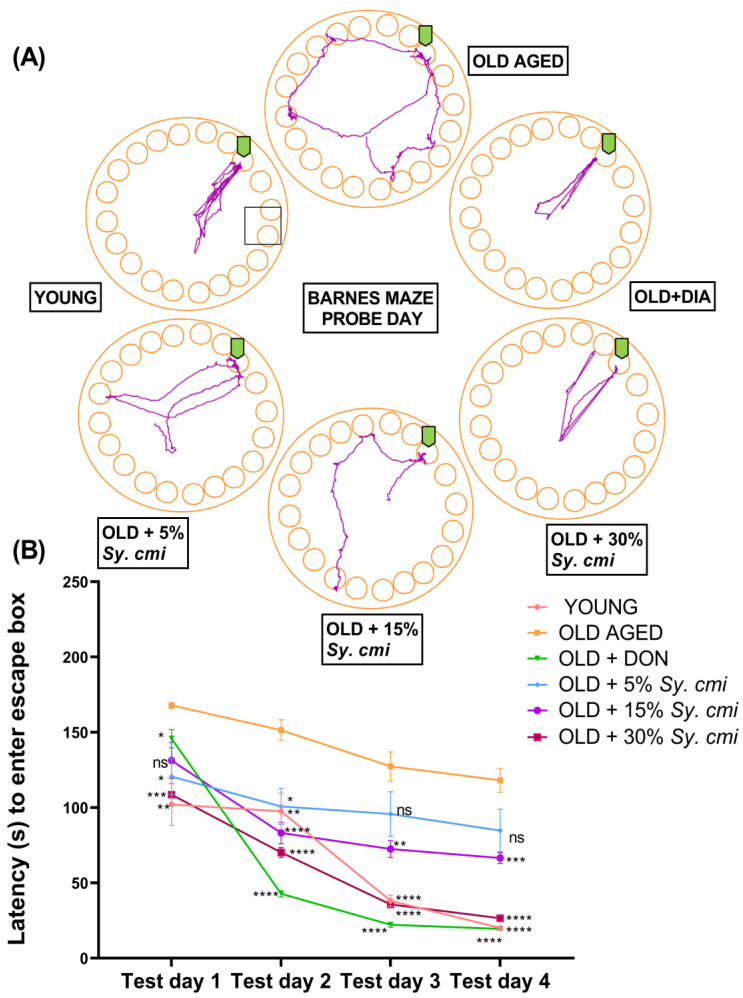
The mice (*n* = 8) supplemented with 5, 15, and 30% dilutions of *Sy. cmi* for 16 months were tested for learning and memory in the Barnes maze test, and the animals’ cognitive ability was tested by monitoring their ability to locate the escape hole in consecutive 5 days. The animals’ activity was expressed as (**A**) representative track plots of animals from the respectively treated group and (**B**) latency to enter the escape hole. The outcomes of all groups were compared with the aged control group and * *p* < 0.05, ** *p* < 0.01, *** *p* < 0.001, **** *p* < 0.0001 were considered statistically significant while ns showed non-sgnificant outcomes for all data presented as mean ± SEM.

The results of two-way ANOVA show that animals of all groups show significant variation in escape latencies (F (42,126) = 51.75, *p* < 0.0001), noted in four trials per day during acquisition days. The aged mice show compromised recalling capability of the target hole, which results in a longer time to reach the escape hole (*p* = 0.007) on the 2nd day of the Barnes maze test, which is further prolonged (*p* < 0.0001) on 4th day. The supplementation with *Sy. cmi* resultes in improved memory of the escape box location. Thus, escape latencies are notably shortened by the 4th day of the test, as 15% and 30% *Sy. cmi* treatment causes significant outcomes with *p* = 0.0008 and *p* < 0.0001, respectively (Figure 8A,B).

**Figure 9:**
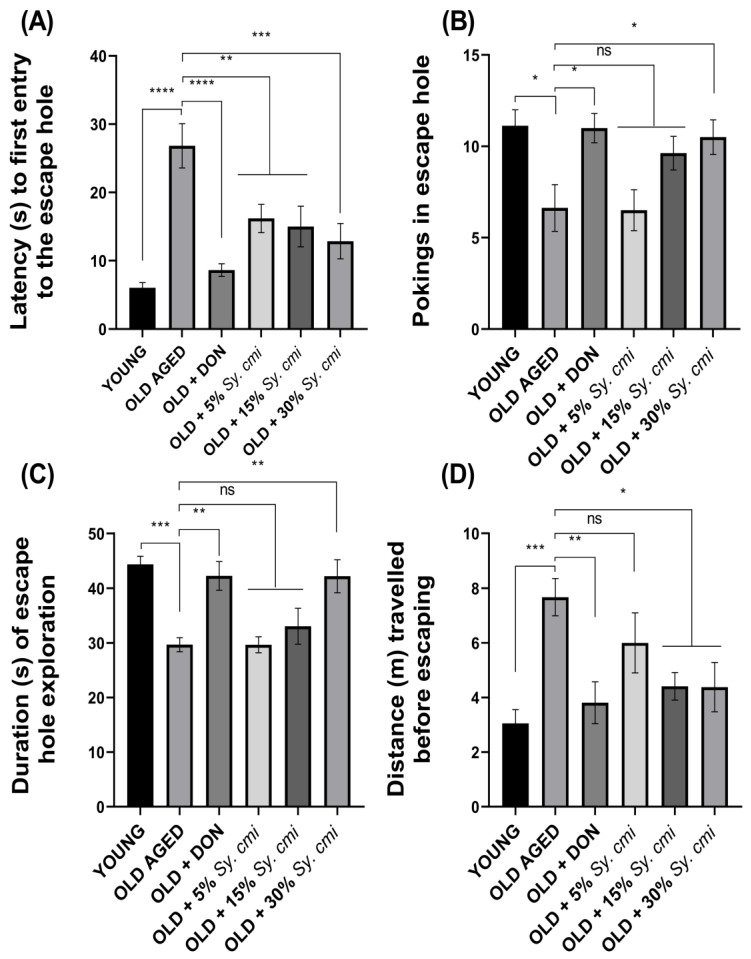
The mice (*n* = 8) supplemented with 5, 15, and 30% dilutions of *Sy. cmi* for 16 months were tested for learning and memory in the Barnes maze test, and the animals’ cognitive ability was tested by monitoring their remembrance of the escape box location on the probe day to note (**A**) latency to first entry in escape hole, (**B**) pokings in escape hole, (**C**) duration of escape hole exploration, and (**D**) distance travelled before escaping, time in escape hole the latency to enter the escape hole. The outcomes of all groups were compared with the aged control group and * *p* < 0.05, ** *p* < 0.01, *** *p* < 0.001, **** *p* < 0.0001 were considered statistically significant while ns showed non-sgnificant outcomes for all data presented as mean ± SEM.

During the probe trial of 90 s duration on the 6th day of the test, significant differences among groups are noted for latency to first poke in the escape hole (F (5,42) = 9.96, *p* < 0.0001) and number of times poking there (F (5,42) = 4.51, *p* = 0.002). As compared to young group, the aged control group has poor remembrance of the escape hole during the probe trial, as they take a longer time to locate the hole, resulting in significant latency to their first poke (*p* < 0.0001) and a reduced amount of head poking (*p* < 0.05) there. However, the aged groups nourished with *Sy. cmi* have concentration-dependent improvement in navigational memory, and the finest outcomes are noted with the 30% *Sy. cmi* group, as their latency to the first poke is significantly shorter (*p* = 0.0005) and the amount of pokes is prominently increased (*p* = 0.038) (Figure 9A,B).

Furthermore, the probe trial has determined the animal’s memory of the escape hole location in terms of the duration of their escape zone exploration and the distance traveled to reach the escape hole, which are notably varied with (F (5,42) = 8.48, *p* < 0.0001) and (F (5,42) = 4.68, *p* = 0.001), respectively. The young group has a better ability to remember the location of the escape hole, as they spend a significantly longer duration in poking the escape hole (*p* = 0.0003) in comparison to aged control group. Similar behavior of longer exploration of escape hole is noted in aged mice supplemented with 30% *Sy. cmi* (*p* = 0.002), as shown in Figure 9C. Moreover, aged mice remain thigmotaxic in the open arena of maze and travel longer distances to reach the escape hole (*p* = 0.0006) as compared to the young group. The nourishment with *Sy. cmi* at concentrations of 15% and 30% causes improved recollection capacity in aged mice, as they travel noticeably shorter distances to reach the escape location with *p* = 0.02 and *p* = 0.018, respectively (Figure 9D).

### 3.9. Biochemical Analysis of Isolated Brains for MDA, SOD, GPx, Catalase, and AChE

**Figure 10:**
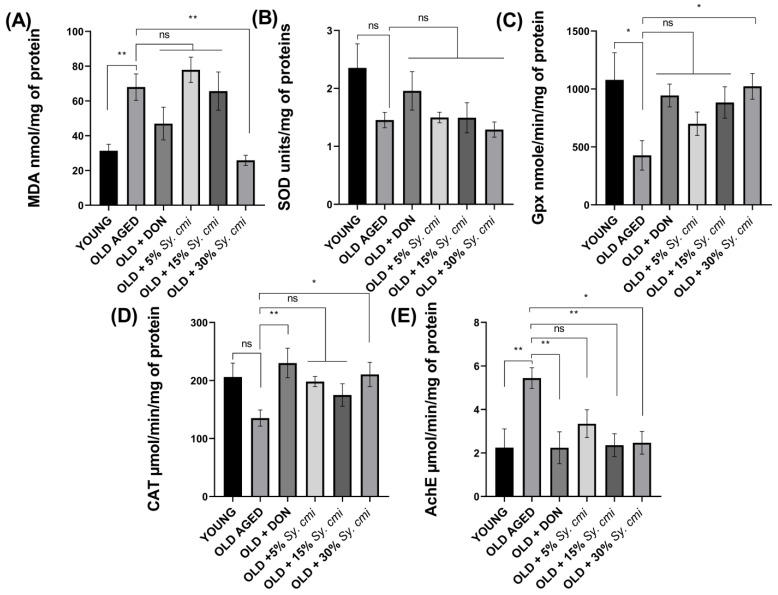
The dissected brains of mice (*n* = 8) supplemented with 5, 15, and 30% dilutions of *Sy. cmi* for 16 months were tested for (**A**) malondialdehyde, (**B**) superoxide dismutase, (**C**) glutathione peroxidase, (**D**) catalase, and (**E**) acetylcholinesterase levels. The outcomes of all groups were compared with the aged control group and * *p* < 0.05, ** *p* < 0.01 were considered statistically significant while ns showed non-sgnificant outcomes for all data presented as mean ± SEM.

The impact of age-related lipid peroxidation, estimated in terms of MDA levels, reveals a prominent difference (F (5,30) = 7.86, *p* < 0.0001) among old untreated and *Sy. cmi*-nourished groups. Post-hoc comparisons indicat a significant increase in MDA levels in aged mice (*p* = 0.0078) in comparison to young brains, while 30% *Sy. cmi* supplementation results in a significant decline in MDA levels (*p* = 0.002), as shown in Figure 10A.

The estimation of endogenously present antioxidant enzyme activity is also an indication of age-related oxidative damage by free radicals. The SOD levels vary non-significantly in groups with (F (5,30) = 2.45, *p* = 0.055) (Figure 10B), while GPx and catalase levels are changed noticeably with (F (5,30) = 2.89, *p* = 0.030) and (F (5,30) = 2.90, *p* = 0.029), respectively. The advanced age leads to a reduction in GPx and catalase enzymes, which are antioxidant defenses, as shown in Figure 10D. In detail, the aged mice brain has significantly reduced GPx (*p* = 0.012), as shown in Figure 10C. However, catalase is reduced non-significantly (*p* = 0.060) as depicted in Figure 10D, in comparison to young mice. The *Sy. cmi* nourishment prevents this deterioration in a concentration-dependent manner, and the most pronounced elevation of GPx (*p* = 0.025) and catalase (*p* = 0.042) is noted with 30% *Sy. cmi*, as compared to aged mice.

The levels of acetylcholinesterase also vary among groups (F (5,30) = 3.86, *p* = 0.008). Significantly higher acetylcholinesterase levels are noted in aged brains (*p* = 0.006), while the 15% and 30% dilutions of *Sy. cmi* show a reduction of acetylcholinesterase enzyme activity with *p* = 0.008 and *p* = 0.011, respectively, and it might be contributing to preventing the breakdown of acetylcholine (Ach), leading to increased cholinergic neurotransmission and memory (Figure 10E).

### 3.10. Molecular Docking

**Figure 11:**
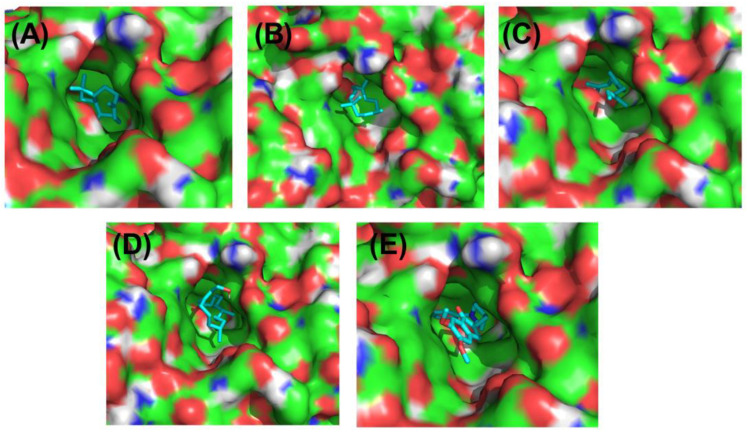
The binding pocket of all investigated compounds, cyan color representing the drugs (**A**–**E**) and green, red, blue, and white colors representing the active pocket site (**A**) caryophyllene, (**B**) humulene, (**C**) beta-farnesene, and (**D**) phytol in comparison to standard drug (**E**) donepezil using target acetylcholinesterase protein 1GQR.

Acetylcholinesterase is an enzyme responsible for breaking down acetylcholine, and donepezil temporarily prevents it from conducting its normal activity. The augmentation of cholinergic transmission due to this enzyme inhibition is assumed to be the principal pharmacological action of the medication, which includes the relief of symptoms associated with Alzheimer’s disease. We show the crystal structure of acetylcholinesterase in its native state (at 2.20 Å resolution) and in its binding with investigated ligands caryophyllene, humulene, beta-farnesene, and phytol, using donepezil as a standard drug (Figure 11). Table 2 shows the binding affinity, hydrogen binding, and hydrophobic and electrostatic interactions with active pocket site amino acids and distances in angstroms.

**Figure 12:**
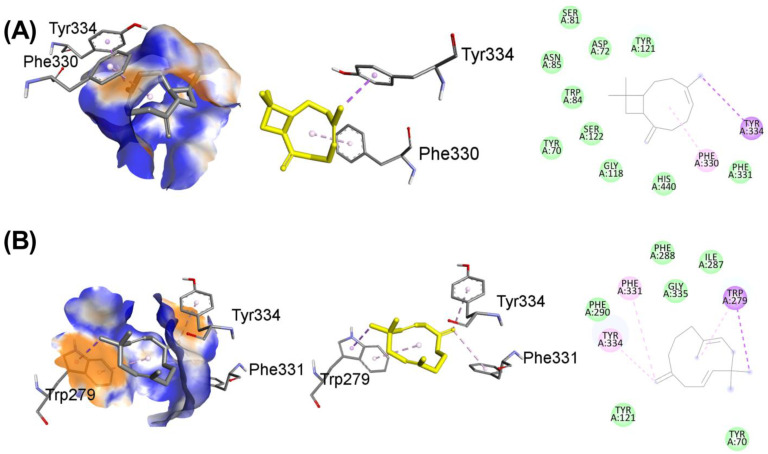
The binding mode with hydrogen acceptance (blue) and hydrogen donor (orange color) surface (leftmost), with 3D interaction having drug in yellow color (middle) and with 2D binding interactions (rightmost) (**A**) caryophyllene and (**B**) humulene using target acetylcholinesterase protein 1GQR. Green colors are indicating hydrogen bonding and weak hydrogen bond interactions, whereas the dark purple color indicates hydrophobic interactions and light purple shows the electrostatic binding linkages.

Molecular virtual screening has helped to identify four compounds with potential hydrogen bond, hydrophobic, and hydrophilic contacts, which are used to assess the protein 3O8Y’s anti-inflammatory binding. Caryophyllene, shown in Figure 12A, has the most promising hydrophobic and hydrophilic interactions. Important chemical interactions involve the following amino acid residues: TYR121(2.89 Å), GLY118(2.79 Å), TRP84(2.63 Å), and SER(3.01 Å). Four hydrogen bonds have been identified to contribute to the stability of the protein–ligand combination. Important active site hydrophobic interactions and electrostatic interactions also play a vital role in this binding, including amino acids TYR334(3.96 Å), PHE331(3.65 Å), ASP(4.30 Å), ASN(4.41 Å), and PHE330(4.29 Å). Compound caryophyllene’s docking score is determined to be −8.1 kcal/mol.

Similarly, the molecular interactions between humulene, shown in Figure 12B, and the following amino acid residues are stronger: PHE290, TYR121, GLY335 having distance 3.73 Å, 2.97 Å, and 3.93 Å. Important amino acid residues of the cholinesterase protein’s active site motifs are revealed to be involved in binding interactions. Additionally, TRP279, PHE331, ILE287, and PHE288 connect through hydrophobic interactions with distances of 3.53 Å, 3.64 Å, 4.56 Å, and 4.89 Å, respectively. Van der Waals interactions, among hydrophobic interactions, play a crucial role in stabilizing the complex. Humulene has a docking score of −8.3 kcal/mol.

**Figure 13:**
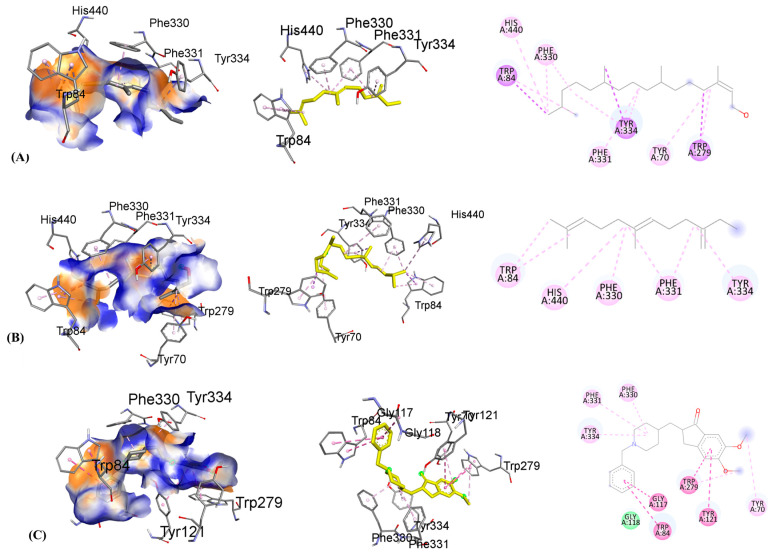
The binding mode with hydrogen acceptance (blue) and hydrogen donor (orange) surface (leftmost), with 3D interaction having drug in yellow color (middle) and with 2D binding interactions (rightmost) (**A**) beta-farnesene, (**B**) phytol, and (**C**) donepezil using target acetylcholinesterase protein 1GQR. The green color indicates hydrogen bonding, whereas the dark purple color is indicating hydrophobic interactions and the light purple color is showing the electrostatic binding linkages.

Another promising lead is beta-farnesene, shown in Figure 13A, which shows strong molecular interactions with amino acid residues in the active site. After the initial round of virtual screening, this drug came in as the third-best possible option. The HIS440, TRP84, TYR334, PHE220, and PHE 331 are amino acid residues that participate in bonding and nonbonding interactions. It has been noticed that two significant hydrophobic linkages with short bond lengths contribute to the complex’s stability. The electronegative oxygen atom of compound C forms a hydrophobic bond with the HIS440 residue of the target protein. In addition, 3.56 Å, 4.12 Å, 4.19 Å, 4.81 Å, and 4.46 Å are the respective molecular binding linkage distances. The beta-farnesene docking score is −7.3 kcal/mol. Similarly, the molecular interactions between phytol, shown in Figure 13B, and the following amino acid residues are stronger: TRP279, TYR334, and TRP84. Additionally, PHE330, PHE331, TYR70, and HIS440 connect through hydrophobic interactions contribute to the conformational stability. Phytol has a docking score of −6.6 kcal/mol.

To co-relate the binding energies and interactions of the caryophyllene, humulene, beta-farnesene, and phytol, the standard drug donepezil is also shown in Figure 13C. The binding active site is found to be the same as the investigated compounds, and the binding interactions of the investigated compounds are a little lower than the donepezil after molecular docking analysis. The following amino acid residues are stronger in donepezil, taking part in excellent binding with cholinesterase enzymes: GLY118, PHE330, TRP84, TYR70, TRP279, TYR121, and TRP84. The donepezil has a docking score of −10.4 kcal/mol.

In addition to molecular docking analysis, the physiochemical properties and structures with chemical properties are shown in the Supplementary Table S1, and absorption, distribution, metabolism, excretion and toxicity (ADMET) studies of the investigated compounds are given in Supplementary Table S2, in comparison to the standard drug donepezil.

## 4. Discussion

Humans have been anciently depending on plants for shelter and food. With time, they have further shifted their attention to seeking remedies for diseases as well [49,50]. According to the United Nations estimation, there are 703 million people over the age of 65 by 2019 globally, which is predicted to reach 1.5 billion by 2050 [51]. Parallelly, the increase in the elderly population has resulted in 50 million cases of dementia worldwide, which might reach 130 million cases by 2050 [52]. With the increased life expectancy, age-associated disorders are becoming a huge burden on the healthcare system and the adoption of remedial dietary strategies is crucial to meet these needs.

The current study has evaluated the impact of prolonged supplementation with *Sy. cmi* on aging-related cognitive decline in mice, as numerous published studies suggest that a diet enriched with antioxidant components may reduce the age-related risk of developing various neurodegenerative diseases, including AD [53,54]. Fruits are enriched with various secondary metabolites of medicinal properties, including phenolic acids and flavonoids as major components [55]. In the present study, the presence of polyphenols and diterpenoids has been revealed in *Sy. cmi*. Polyphenols are one of the vital phytoconstituents owned by plants and their fruits, which reduce the aging-associated increased oxidative stress and lipid peroxidation in the brain, hence preventing the progression of pathological changes, leading to neurodegeneration [56].

The supplementation with 5, 15, and 30% of *Sy. cmi* dilutions for 16 months shows that this dietary add-up has had beneficial effects on age-related anxiety-like behavior in mice. The results of OFT show that aging causes a reduction in locomotion with an increase in anxiety as aged control mice spend more time in protected and dark zones of the experimental maze. Similarly, age-associated anxiety has been noticed in the aged control group during the LD and EPM tests. Similar age-related behavior alteration outcomes have been observed by Shoji et al. in a series of behavioral tasks, including light/dark transition and decreased locomotor activity in the OFT and EPM tests [23]. Exposure to the novel environment might result in higher cortisone levels in aged mice, which could be the reason for their anxiety-like behaviors. However, earlier studies have found some contradictory results on age-related changes in anxiety-like behaviors, as a previous study examining the animals’ preference for the central zone reports no impact of advancing age on rodent anxiety [57]. The polyphenolic compounds possessed by *Sy. cmi* might be playing a role in modulating the GABAergic neurotransmission levels, leading to neuronal inhibition and reduced anxiety in *Sy. cmi*-treated groups [58]. In a previous study, the ameliorative effect of polyphenol-rich *Aronia melanocarpa* berries on anxiety has been noted in rats consuming berry juice for 1 month [59]. In the same way, rats given epigallocatechin-3-gallate, a key polyphenol in tea, for several days show a strong anxiolytic effect in the open field and light/dark tests [60].

To recompense the cholinergic neuronal death, acetylcholinesterase inhibitors are dominantly implicated as the palliative treatment of AD, as these agents restore the synaptic levels of acetylcholine. Further, it is also reported that acetylcholinesterase itself is also involved in the pathogenesis of Alzheimer’s disease, as it directly interacts with amyloid β, resulting in increased deposition of this peptide into insoluble plaques [61]. Thus, properly designed anticholinesterases might work as disease-modifying agents. In this study, the isolated aged untreated mice brains have shown elevated AChE activity that contributes to diminished cholinergic neurotransmission and associated cognitive impairment in these animals, as appropriate cholinergic neurotransmission is fundamental for the formation of memory. However, the mice chronically nourished with *Sy. cmi* have reduced AChE levels that might be associated with their protection from age-associated memory deficit. *Sy. cmi* nourishment results in better alterations in the T-maze and improved recognition of the novel arm in the Y-maze. Further, these mice interact with the novel object for a longer duration and their escape latencies are reduced in Barnes maze tests, further authenticating that *Sy. cmi* exerts protection from the detrimental effects of age on the brain. Flavonoids are the phytoconstituents known to elevate the acetylcholine levels in the hippocampus by inhibiting AChE. Additionally, flavonoids bind to and activate cAMP response element-binding proteins (CREBs), which increase neurotransmission and information flow and cause the overexpression of genes that affect memory. Furthermore, these phytoconstituents may prevent the activation of caspase-3, which would have antiapoptotic effects and protect neurons [62]. Moreover, they are known to stimulate the production of neurotrophic factors, which increase neuronal proliferation, and as a result, they may reverse anxiety and aging-impaired adult hippocampus neurogenesis [63]. Thus, the outcomes revealing reduced dementia-like signs in aged mice might be attributed to adding *Sy. cmi* to the normal rodent feed as polyphenol supplementation halts the memory deficit in aged mice models. In a recent study, 5-week-old mice provided with a diet added with coffee polyphenols for 5 months have had improved cognitive behavior and reduced deposition of amyloid β plaques in the hippocampus [64]. Another study correspondingly reports feeding the mice with a polyphenol-rich extract from grape and blueberry ameliorates age-associated cognitive impairment [65].

The brain is highly susceptible to oxidative stress due to its high lipid content, increased oxygen requirement, and diffident antioxidant defenses. This intrinsic oxidative vulnerability may be a probable underlying cause of neuropsychiatric illnesses and other neurological diseases [66]. With aging, the membrane fatty acid composition is changed and the generation of free radicals prevails over the endogenous defense mechanisms, leading to lipid peroxidation. Reduced membrane fluidity and damage to membrane proteins caused by oxidative damage to neuronal membrane lipids render receptors, enzymes, and ion channels inactive. As a result, oxidative stress can affect brain activity in general, as well as neurotransmission and neuronal function [67].

The oxidative profiling of isolated brains in terms of lipid peroxidation levels describes a significant increase in MDA levels in the older group compared to those aged mice supplemented with *Sy. cmi* fruit pulp exudates. Similarly, SOD, GPX, and CAT activities are higher in these *Sy. cmi*-treated animals in comparison to aged mice, suggesting that reduced oxidative stress may be linked to protection from cognitive deterioration. These findings imply that berries’ antioxidants may be able to lessen the brain’s oxidative damage and regulate the cholinergic system. Studies have shown that phytoconstituents with antioxidant properties protect from neurological illnesses, as Imran et al. reports that the brains of rats chronically treated with phenols containing berries of Grewia asiatica have increased GPx and SOD levels and decreased MDA levels.

The chemical characterization of *Sy. cmi* shows the occurrence of caryophyllene and humulene, which are naturally present sesquiterpenes in plants. In the present study, these phytocompounds show strong interactions with the active site of acetylcholinesterase enzyme, and that might be the mechanism through which *Sy. cmi* exertw protective effects against age-associated cognitive decline in mice. The findings are strengthened by Lindsey et al., in which sub-chronically administered β-caryophyllene at a dose of 100–300 mg/kg reverses age-related cognitive impairment in aged mice through its anti-inflammatory effects [68]. In another study, Sudeep et al. encourages the use of β-caryophyllene as a dietary supplement for memory enhancement, as caryophyllene (50–100 mg/kg) treatment for 2 weeks protects the mice from scopolamine-induced amnesia through modulating the neuroinflammatory markers [69]. Further, Ojha et al. also reports that naturally occurring β-caryophyllene works as a CB2 receptor agonist and exerts neuroprotection by attenuating glial activation, neuroinflammation, and oxidative stress [70], leading to the reversal of amyloid-induced memory deficit [71]. The CB2 receptor is a component of the endogenous cannabinoid system and plays a role in Alzheimer’s disease (AD), as the levels of CB2 receptors are increased in AD brains and are correlated with β-amyloid plaque deposition [72]. In a molecular docking study, we have noted that caryophyllene shows promising hydrophobic and hydrophilic interactions with the acetylcholinesterase enzyme. Further, another sesquiterpene, farnesene, is owned by *Sy. cmi* fruit, which might be attributed to its memory-improving characteristics, as Arsalan et al. reports the neuroprotective effects of this sesquiterpene in an in vitro experimental model of Alzheimer’s disease [73].

The addition of Β-farnesene in the diet as a supplementary nutrient has been reported to exert significant benefits in Alzheimer’s disease. This sesquiterpene has also been reported to ameliorate the cytotoxicity in SH-SY5Y cell culture, thus protecting it from b-amyloid toxicity. Further, the acetylcholinesterase activity in patients with Alzheimer’s disease has been improved by farnesene application [74]. Another phytocompound detected in *Sy. cmi* is phytol, a diterpene alcohol that has been known for anti-cholinesterase and antioxidant properties, and its protective effect against scopolamine-induced amnesia in Wistar rats has also been reported previously [75].

## 5. Conclusions

The outcomes of the current study reveal that the fruit pulp of *Syzygium cumini* (L.) Skeels is rich in polyphenols and terpenoids. Further, the prolonged supplementation of young mice with *Sy. cmi* shows protection from anxiety and age-related cognitive deficit in aged mice. The biochemical analysis of isolated brains shows reduced oxidative stress and acetylcholinesterase activity in *Sy. cmi*-treated mice that might be due to antioxidant effects of polyphenolic phytoconstituents, as well as strong interactions of detected terpenoids with the active site of acetylcholinesterase. However, the molecular mechanisms behind the age-associated neurological benefits of *Sy. cmi* supplementation are required to be thoroughly explored in the future.

## Figures and Tables

**Table 1:** Phytocompounds detected by GC-MS in *Syzygium cumini* (L.) Skeels.

Serial No.	Phytocompounds	RT (Retention Time)	Area%
1	trans-beta-Ocimene	2.271	0.37
2	5-Hydroxymethylfurfural	7.449	18.76
3	Caryophyllene	15.098	0.80
4	Humulene	17.076	0.38
5	beta-Farnesene	17.508	0.43
6	Dodecanoic acid	20.579	0.46
7	Oleic Acid	30.500	1.13
8	Phytol	30.139	0.18

**Table 2:** Binding affinity (kcal/mol), hydrogen binding, and hydrophobic and electrostatic interactions with distances in Angstrom for investigated ligands using acetylcholinesterase protein having the PDB ID: 1GQR.

Ligands with 1GQR	Binding Affinity, ΔG (kcal/mol)	Hydrogen-Binding Interaction, Residue (Distance Å)	Hydrophobic Interaction, Residue (Distance Å)	Electrostatic Interaction, Residue (Distance Å)
Caryophyllene	−8.1	TYR121(2.89) GLY118(2.79) TRP84(2.63) SER122(3.01)	TYR334(3.96) PHE331(3.65) ASP72(4.30) ASN85(4.41)	PHE330(4.29)
Humulene	−8.3	PHE290(2.73) TYR121(2.97) GLY335(3.93)	TRP279(3.54) PHE331(3.64) ILE287(4.56) PHE288(4.89)	TYR334(4.12)
beta-Farnesene	−7.3		HIS440(3.56) TRP84(4.12) TYR334(4.19)	PHE330(4.81) PHE331(4.46)
Phytol	−6.6		TRP279(3.63) TYR334(3.93) TRP84(3.72)	PHE330(4.26) PHE331(4.14) TYR70(4.73) HIS440(5.01)
Donepezil	−10.4	GLY118(2.38)	PHE330(3.96) TRP84(3.63) TYR70(4.98) TRP279(4.57) TYR121(4.11) TRP84(4.72)	TRP279(5.36) GLY117(5.82) PHE331(5.23) TYR334 (5.21) TRP279(5.04)

## Data Availability

The data presented in this study are available on request from the corresponding authors.

## Supplementary Materials

The following supporting information can be downloaded at: https://www.mdpi.com/article/10.3390/nu15030666/s1, Table S1: Physiochemical properties, 2D(Chemdraw) and 3D (Chemdraw 3D), and chemical descriptors of investigated compounds A–E; Table S2: Adsorption, Distribution, Metabolism, Excreaction and Toxicological properties of the investigated compounds (A–D) in comparison to donepezil (E) drug.
