# Supplementary material for: Long-Term Supplementation of Syzygium cumini (L.) Skeels Concentrate Alleviates Age-Related Cognitive Deficit and Oxidative Damage: A Comparative Study of Young vs. Old Mice
Source: Nutrients. 2023 Jan 28;15(3):666. doi: 10.3390/nu15030666 (PMC9921576; doi:10.3390/nu15030666)
Supplement: Supplementary file 1 [file nutrients-15-00666-s001.zip › nutrients-2183435-supplementary.pdf]

nutrients

MDPI

Long-term Supplementation of *Syzygium cumini* (L.) Skeels Concentrate Alleviates Age-related Cognitive Deficit and Oxidative Damage: A Comparative Study of Young vs Old Mice

Nosheen Malik [1], Sana Javaid [1,2], Waseem Ashraf [1], Farhan Siddique [3], Muhammad Fawad Rasool [4], Faleh Alqahtani [5,*], Tanveer Ahmad [6], Muhammad Asad Abrar [7] and Imran Imran [1,*]

Supplementary Materials

Supplementary Table S1. Physiochemical properties, 2D(Chemdraw) and 3D (Chemdraw 3D), and chemical descriptors of investigated compounds A–E.

Compound Name	2D Structure	Chemical Properties	Chemical Analysis	3D Structure
Caryophyllene (A)		Boiling Point: 576.5 [K]<br>Melting Point: 323.21 [K]<br>Critical Temp: 737.59 [K]<br>Critical Pres: 20.27 [Bar]<br>Critical Vol: 716.5 [cm3/mol]<br>Gibbs Energy: 196.63 [kJ/mol]<br>Log P: 4.48<br>MR: 67.73 [cm3/mol]<br>Henry's Law: -1.45<br>Heat of Form: -112.68 [kJ/mol]<br>tPSA: 0<br>CLogP: 6.453<br>CMR: 6.6238<br>LogS: -4.156<br>pKa: N/A	Chemical Formula: $C_{15}H_{24}$<br>Exact Mass: 204.19<br>Molecular Weight: 204.36<br>m/z: 204.19 (100.0%), 205.19 (16.5%), 206.19 (1.2%)<br>Elemental Analysis: C, 88.16; H, 11.84	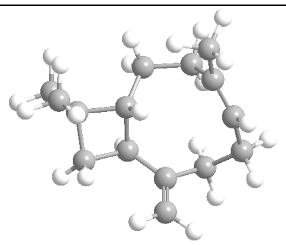
Humulene (B)		Boiling Point: 586.4 [K]<br>Melting Point: 299.71 [K]<br>Critical Temp: 755.3 [K]<br>Critical Pres: 20.53 [Bar]<br>Critical Vol: 722.5 [cm3/mol]<br>Gibbs Energy: 137.25 [kJ/mol]<br>Log P: 4.83<br>MR: 71.02 [cm3/mol]<br>Henry's Law: -1.75<br>Heat of Form: -125.84 [kJ/mol]<br>tPSA: 0<br>CLogP: 6.773<br>CMR: 6.7758<br>LogS: -4.195<br>pKa: N/A	Chemical Formula: $C_{15}H_{24}$<br>Exact Mass: 204.19<br>Molecular Weight: 204.36<br>m/z: 204.19 (100.0%), 205.19 (16.5%), 206.19 (1.2%)<br>Elemental Analysis: C, 88.16; H, 11.84	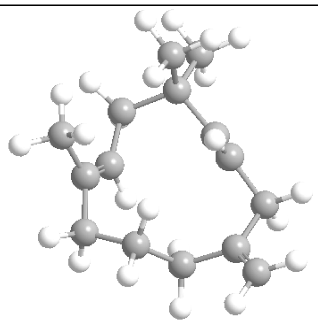
(E)-.beta.-Farnesene (C)		Boiling Point: 547.44 [K]<br>Melting Point: 204.51 [K]<br>Critical Temp: 705.83 [K]<br>Critical Pres: 16.04 [Bar]<br>Critical Vol: 819.5 [cm3/mol]<br>Gibbs Energy: 298.05 [kJ/mol]<br>Log P: 5.13<br>MR: 72.95 [cm3/mol]<br>Henry's Law: -1.12<br>Heat of Form: -22.43 [kJ/mol]<br>tPSA: 0<br>CLogP: 6.787<br>CMR: 7.0582<br>LogS: -3.608<br>pKa: N/A	Chemical Formula: $C_{15}H_{26}$<br>Exact Mass: 206.20<br>Molecular Weight: 206.37<br>m/z: 206.20 (100.0%), 207.21 (16.5%), 208.21 (1.3%)<br>Elemental Analysis: C, 87.30; H, 12.70	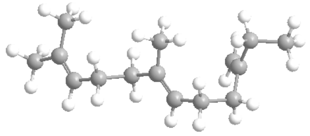

Phytol (D)	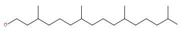	Boiling Point: 752.1 [K]<br>Melting Point: 311.44 [K]<br>Critical Temp: 777.23 [K]<br>Critical Pres: 11.37 [Bar]<br>Critical Vol: 1137.5 [cm3/mol]<br>Gibbs Energy: 45.05 [kJ/mol]<br>Log P: 6.96<br>MR: 97.17 [cm3/mol]<br>Henry's Law: 1.6<br>Heat of Form: -516.77 [kJ/mol]<br>tPSA: 20.23<br>CLogP: 8.483<br>CMR: 9.5811<br>LogS: -5.139<br>pKa: 14.594	Chemical Formula: $C_{20}H_{42}O$<br>Exact Mass: 298.32<br>Molecular Weight: 298.56<br>m/z: 298.32 (100.0%), 299.33 (22.2%), 300.33 (2.5%<br>Elemental Analysis: C, 80.46; H, 14.18; O, 5.36	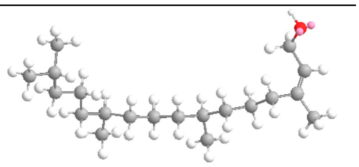
Donepezil (E)	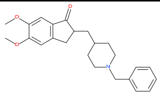	Boiling Point: 964.14 [K]<br>Melting Point: 624.13 [K]<br>Critical Temp: 952.21 [K]<br>Critical Pres: 14.84 [Bar]<br>Critical Vol: 1122.5 [cm3/mol]<br>Gibbs Energy: 222.62 [kJ/mol]<br>Log P: 4.01<br>MR: 113.97 [cm3/mol]<br>Henry's Law: 10.3<br>Heat of Form: -301.37 [kJ/mol]<br>tPSA: 38.77<br>CLogP: 4.59759<br>CMR: 11.1212<br>LogS: -4.609<br>pKa: 9.287	Chemical Formula: $C_{24}H_{29}NO_3$<br>Exact Mass: 379.21<br>Molecular Weight: 379.50<br>m/z: 379.21 (100.0%), 380.22 (26.4%), 381.[illegible]<br>Elemental Analysis: C, 75.96; H, 7.70; N, 3.69	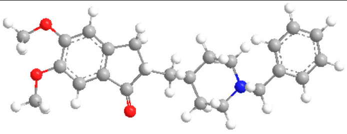

K: kelvin, cm3/mol: centimeter cube in one mole, kJ/mol: kilojoules in one mole.

Supplementary Table S2. Adsorption, Distribution, Metabolism, Excretion and Toxicological properties of the investigated compounds (A–D) in comparison to donepezil (E) drug.

ADMET	A	B	C	D	Donepezil (E)
**Physiochemical Properties**					
Formula	$C_{15}H_{24}$	$C_{15}H_{24}$	$C_{15}H_{26}$	$C_{20}H_{40}O$	$C_{24}H_{29}NO_3$
Molecular Weight	204.35	204.35	206.37	296.53	379.49
No.of Heavy Atoms	15	15	15	21	28
No.arom.Heavy Atoms	0	0	0	0	12
Fraction Csp3	0.73	0.6	0.6	0.9	0.46
No. of rotatable bonds	0	0	7	13	6
No.of H.bond Acceptor	0	0	0	0	4
No. of H.bod donors	0	0	0	0	0
Molar Refractivity	68.78	70.42	72.8	98.94	115.31
Topological polar surface Area (TPSA) A^2	0	0	0	20.23	38.77
**Lipophilicity (Log $P_{o/w}$)**					
iLOGP	3.29	3.3	4.02	4.71	3.92
XLOGP3	4.38	4.79	6.21	8.19	4.28
WLOGP	4.73	5.04	5.43	6.36	3.83
MLOGP	4.63	4.53	4.93	5.25	3.06
Silicos-IT Log P	4.19	4.19	4.94	6.57	4.91
Consensus Log P (average of all above predictions)	4.24	4.37	5.1	6.22	4
**Water Solubility**					
Log S (ESOL)	−3.87	−4.12	−4.57	−5.98	−4.81
Solubility (mg/ml)	2.78E-02	1.53E-02	5.56E-03	3.10E-04	5.87E-03
Class	Soluble	Moderately soluble	Moderately soluble	Moderately soluble	Moderately soluble
Log S (Ali)	−4.1	−4.52	−6	−8.47	−4.81
Solubility (mg/ml)	1.64E-02	6.15E-03	2.09E-04	9.94E-07	5.92E-03
Class	Moderately soluble	Moderately soluble	Moderately soluble	Poorly soluble	Moderately soluble
Log S (SILICOS-IT)	−3.77	−3.77	−4.08	−5.51	−6.9
Solubility (mg/ml)	3.49E-02	3.49E-02	1.70E-02	9.06E-04	4.78E-05
Class	Soluble	Soluble	Moderately soluble	Moderately soluble	Poorly soluble
**Pharmacokinetics**					
GI absorption	medium	medium	Low	Low	High
BBB permeant	No	No	No	No	Yes
P-gp substrate	No	No	No	Yes	Yes
CYP1A2 inhibitor	No	No	Yes	No	No
CYP2C19 inhibitor	Yes	No	No	No	No
CYP2C9 inhibitor	Yes	Yes	Yes	Yes	No
CYP2D6 inhibitor	No	No	No	No	Yes
CYP3A4 inhibitor	No	No	No	No	Yes
Log Kp (skin permeation) cm/s	−4.44	−4.15	−3.15	−2.29	−5.58
**Drug Likeness**					
Lipinski Violation	1	1	1	1	0
Ghose Violation	0	0	0	1	0
Veber Violation	0	0	0	1	0
Egan Violation	0	0	0	1	0
Muegge Violation	1	1	2	2	0
Bioavailability	0.55	0.55	0.55	0.55	0.55
**Medicinal Chemistry**					

Pan Assay Interference Structure (PAINS)	0	0	0	0	0
Brenk	1	1	1	1	0
Leadlikeness	2	2	2	2	2
Synthetic Accessibility	4.51	3.78	3.18	4.3	3.36

XLOGP3: pure atom-additive model, LOGP: octanol-water partition coefficient, Log S: water solubility of a drug, CYP: Cytochrome P450, Log Kp: Skin permeability.
